# Neuromotor Development in the *Shank3* Mouse Model of Autism Spectrum Disorder

**DOI:** 10.3390/brainsci12070872

**Published:** 2022-06-30

**Authors:** Miriam Pillerová, Diana Drobná, Jakub Szabó, Emese Renczés, Veronika Borbélyová, Daniela Ostatníková, Peter Celec, Ľubomíra Tóthová

**Affiliations:** 1 Faculty of Medicine, Institute of Molecular Biomedicine, Comenius University in Bratislava, 811 08 Bratislava, Slovakia; miriam.pillerova@gmail.com (M.P.); drobnadiana@gmail.com (D.D.); szabo.jakub4@gmail.com (J.S.); renczes.emese@gmail.com (E.R.); borbelyova.veronika88@gmail.com (V.B.); petercelec@gmail.com (P.C.); 2 Faculty of Medicine, Institute of Physiology, Comenius University in Bratislava, 813 72 Bratislava, Slovakia; daniela.ostatnikova@fmed.uniba.sk; 3 Faculty of Medicine, Institute of Pathophysiology, Comenius University in Bratislava, 811 08 Bratislava, Slovakia

**Keywords:** animal model, autism, developmental delay, early diagnosis, neonatal development

## Abstract

Although autism spectrum disorder (ASD) is mainly characterized by developmental delay in social and communication skills, it has been shown that neuromotor deficits are an early component of ASD. The neuromotor development of B6.129-*Shank3*^tm2Gfng/J^ (*Shank3B*^−/−^) mice as an animal model of autism has not been analyzed yet. The aim of this study was to compare the early neuromotor development of *Shank3B*^−/−^ to wild-type mice. The mice underwent a multitude of neurodevelopmental tests and observations from postnatal day 1 (PND = 1) to weaning. *Shank3B*^−/−^ mice opened their eyes later than their wild-type litter mates (*p* < 0.01). *Shank3B*^−/−^ mice were also slower in the negative geotaxis test from PND = 13 to PND = 16 (*p* < 0.001) in both sexes. The results of this study indicate neurodevelopmental deficits in *Shank3B*^−/−^ mice. The test is partially dependent on truncal motor control, and these lines of evidence suggest a phenotype of developmental hypotonia, which corresponds with the phenotypes seen in patients with Phelan-McDermid Syndrome. There was no observable effect of sex in any of the tests. There were no observed differences in upper and lower incisor eruption, ear unfolding, air righting, surface righting and ear twitch reflexes. Further studies should prove whether the delay in neuromotor development is linked to social or communication deficits, and thus, whether it may serve as an early indicator of autistic-like phenotype in mice.

## 1. Introduction

Autism Spectrum Disorder (ASD) is a general term used for a heterogeneous group of neurodevelopmental disorders. The main behavioral symptoms of ASD are communication deficits, impaired social interaction, and repetitive behavior. Neurodevelopmental delay and motor dysfunction are also common features of ASD, and may be the first signs prior to behavioral manifestation [1]. Poor coordination and trouble with learning of complex motor skills are the most typical disabilities related to impaired neuromotor development [2]. These characteristics could play an important role as markers for early ASD diagnosis, and better understanding of them may lead to novel treatment targets and clinical stratification [3].

Multiple factors play a role in the etiology of ASD. However, the genetic background of ASD is undeniable. The heritability of ASD is almost 90% [4,5]. There are hundreds of known genes associated with ASD in humans. One of the best-described genes is a highly conserved SH3 and multiple ankyrin repeat domains 3 gene (*Shank3*) [6]. The SHANK3 protein plays an important role in the postsynaptic membrane at glutamatergic synapses as a scaffolding protein. It is crucial for the zinc-sensitivity signaling system and regulates postsynaptic excitatory neurotransmission [7]. Deletion or other mutations of the terminal end of the *Shank3* gene lead to a series of different but related symptoms, including a wide range of intellectual, and behavioral deficits [8]. In humans, mutations in *Shank3* may result in Phelan-McDermid syndrome, which is characterized by developmental delay, absent or delayed speech, hypotonia, intellectual disability, and in most of the cases, the core symptoms of autism (repetitive behavior, social and communication deficits). Furthermore, mutations in the *Shank3* gene can cause not only ASD-like phenotype [9,10] but also symptoms of other disorders such as schizophrenia and Rett syndrome [11,12] About 75% of people with heterozygous mutation in *Shank3* gene have been diagnosed with ASD [13]. However, the prevalence of this mutation in ASD patients is only approximately 0.7–2.3% [14,15]. Based on previous results showing neurodevelopmental delay in children with autism, we expected to observe a similar phenotype in animals with the *Shank3* gene mutation [16,17].

Since ASD is a neurodevelopmental disorder, to understand its etiopathogenesis, the symptoms should be examined as early as in the perinatal period of development. Therefore, in this study, we focused on neurodevelopmental milestones in the widely used genetic model of ASD, *Shank3B*^−/−^ mice. Our main goal was to investigate whether infant *Shank3B*^−/−^ female and male mice have impaired neurodevelopment causing sensory-motor dysfunctions in comparison to wild-type litter mates. In addition, we aimed to examine whether male sex delays neurodevelopment in *Shank3B*^−/−^ mice.

## 2. Methods

This study was conducted according to the EU Guidelines for scientific experimentation on animals (Directive 2010/63/EU), after the approval of the protocol by the State Veterinary and Food Administration of the Slovak Republic and the Ethical Committee of the Institute of Pathophysiology in Bratislava, Slovak Republic. Approval number: 05/2017/SKU11016.

### 2.1. Animals

Pairs of heterozygous knockout B6.129-*Shank3*^tm2Gfng/J^ (also known as *Shank3B*^−/−^) were obtained from the Jackson laboratory (JAX^®^ Laboratory, USA). All animals (*Shank3B*^−/−^: ♀ *n* = 13, ♂ *n* = 10; Wild-Types: ♀ *n* = 29, ♂ *n* = 15) were housed with their mothers until weaning and weighed every day before testing. Heterozygotes were not used in this study; they were only used for breeding. Harem scheme for breeding was employed (1 male and 3 females per each cage). The animals were kept under stable room temperature (21–24 °C), humidity (55–65%), and 12-h light/dark cycle conditions.

### 2.2. Neurodevelopment

**Figure 1:**
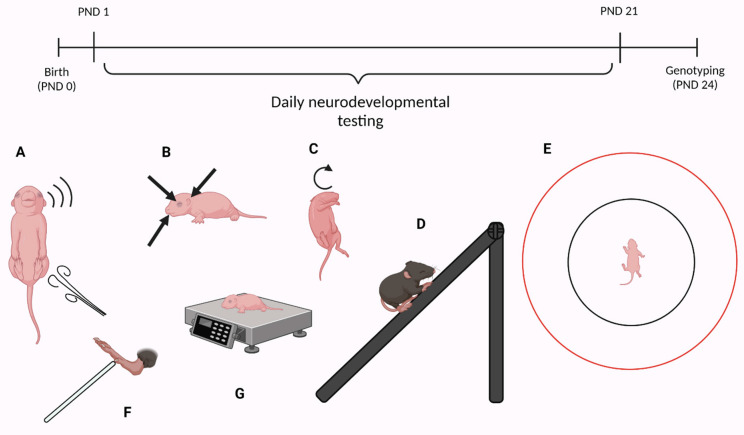
Timeline of the study including schematic for each measurement carried out daily. (**A**) Auditory and tactile startle. (**B**) Ear twitch reflex, ear unfolding, eye opening, upper/lower incisor eruption. (**C**) Surface and air righting. (**D**) Negative geotaxis. (**E**) Gait, walking initiation. (**F**) Forelimb grasp. (**G**) Body weight. Created with BioRender.com (accessed on 13 May 2022).

The physical and sensory-motor development of the pups were assessed from the first postnatal day (PND) until weaning (PND = 21) using a battery of tests adapted from the Fox scale [18,19]. The tests were carried out in the morning (from 8–10 am). The parameters were divided into three categories: 1. landmarks of development, 2. reflexes, 3. motor skills (Figure 1), and were assessed as described below.

### 2.3. Physical and Morphological Landmarks of Development

*Ear unfolding*: The PND when both pinnae were completely separated from the cranium of the pup was recorded. *Eye opening*: The PND when both eyes of the pup were open was recorded. *Upper/lower incisor eruption*: The PND when upper/lower teeth were observable with naked eye was recorded.

### 2.4. Reflexes

*Surface righting*: The pup was placed on its back. The time until it fully turned over on its belly was recorded. The trial was stopped after 60 s, even if the task was not successfully fulfilled. *Air righting* (from PND = 4): The pup was held upside-down at a height of 25 cm and dropped onto a heavily padded surface. The task was positively evaluated if the mice successfully righted themselves during the fall and landed on all four paws. *Forelimb grasp*: The paw of the pups was stroked on the underside with a blunt toothpick. The presence of grasping reflex was recorded. *Ear twitch reflex*: The ear was gently stroked with a cotton swab. The day when the reflex was first observed was recorded. *Auditory startle*: The presence of the startle reflex of pups to an acoustic stimulus, a clicking noise, was evaluated. *Tactile startle*: The presence of the startle reflex of mice to a gentle puff of warm air was observed.

### 2.5. Motor Skills

*Negative geotaxis*: This test represents an innate postural response of rodents to detection of gravitational stimuli. The tested pup rotated 180° from a head-down position to a head-up position. The pup was placed facing head down on a mesh-covered inclined plane at a 30° angle. The latency time to turn and climb up was recorded with a maximum of 60 s. *Gait*: The tested pup was placed in the center of a drawn circle with a radius of 5 cm. The time when the pup crossed the outer border of the circle with all four limbs, as well as the first day when the tested pup successfully performed the task were recorded. The pup failed the test if it was not able to perform the task within 60 s. *Walking initiation*: The tested pup was placed in the center of a circle with a radius of 15 cm. The time when the pup crossed the outer border of the circle with all four limbs was measured with a maximum time limit of 60 s. The PND of the first successful attempt was recorded.

### 2.6. Statistical Analysis

Data were analyzed by two-way analysis of variance (ANOVA) to test the effects of genotype (*Shank3B*^−/−^, wild-type) and sex. Repeated measures two-way ANOVA test (constant factor being genotype, continuous variable being time) was used when the performance in surface righting, negative geotaxis, gait, and walking initiation was assessed on several consecutive days. Bonferroni multiple comparison test was performed if statistical significance (*p* < 0.05) was noted by ANOVA. Results are expressed as mean + SEM.

## 3. Results

### 3.1. Body Weight

**Figure 2:**
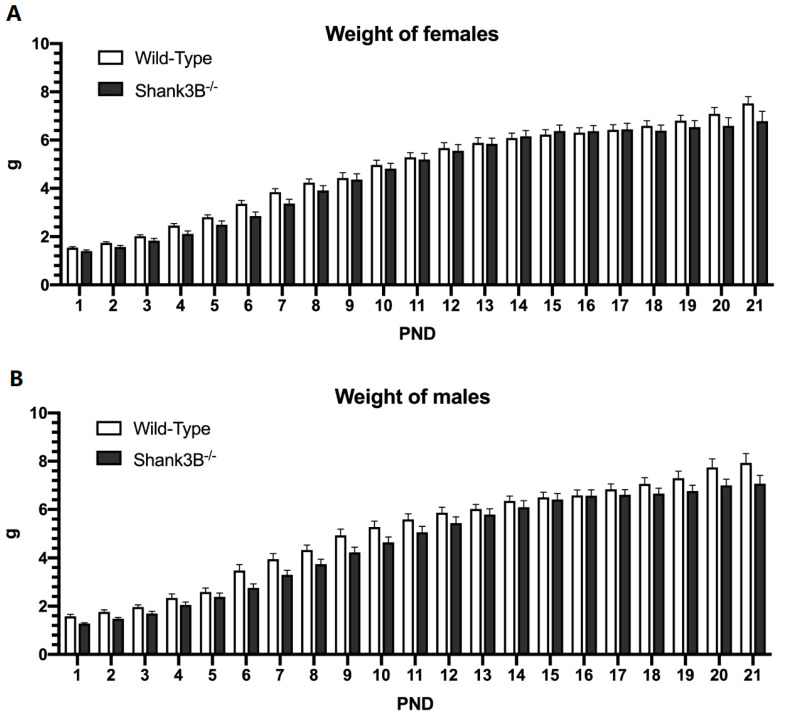
Body weight: (**A**) Females and (**B**) males from PND = 1 to PND = 21. PND = postnatal day; Data are shown as mean + SEM. *Shank3B*^−/−^: ♀ *n* = 13, ♂ *n* = 10; Wild-Types: ♀ *n* = 29, ♂ *n* = 15.

Significant differences in body weight were not observed between genotypes in either females (F(1,28) = 0.61, *p* = 0.44, Figure 2A) or males (F(1,23) = 2.13, *p* = 0.16; Figure 2B).

### 3.2. Physical and Morphological Landmarks of Development

**Figure 3:**
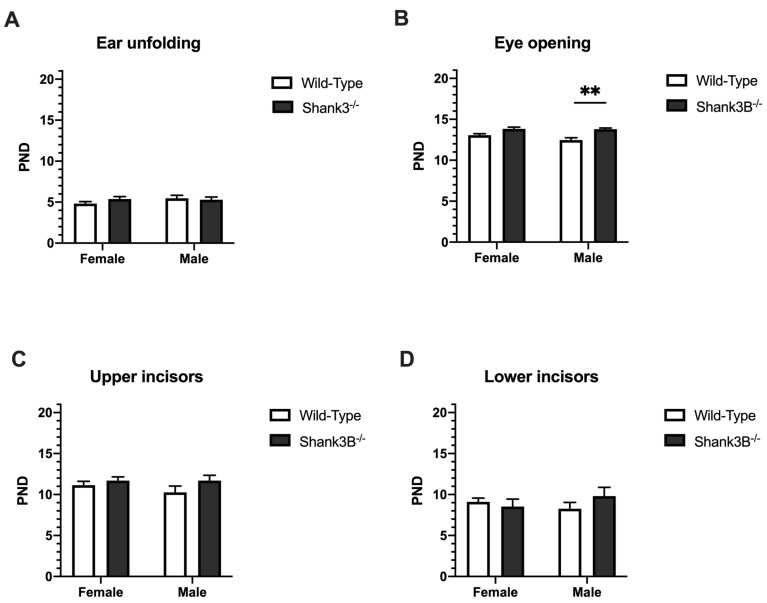
Physical and morphological landmarks of development: (**A**) Ear unfolding. (**B**) Eye opening. (**C**,**D**) Upper and lower incisors eruption, respectively. PND = postnatal day; ** denotes *p* < 0.01. Data are shown as mean + SEM *Shank3B*^−/−^: ♀ *n* = 13, ♂ *n* = 10; Wild-Types: ♀ *n* = 29, ♂ *n* = 15.

There were no significant differences in the development of physical landmarks such as ear unfolding and incisor eruption in *Shank3B*^−/−^ mice in comparison to their wild-type litter mates in either sex (*p* > 0.05, Figure 3). The ears of the animals were unfolded before PND = 9 and there were no effects of sex (F(1,63) = 0.07, *p* = 0.40, Figure 3A) or genotype (F(1,63) = 0.35, *p* = 0.55, Figure 3A), nor a significant interaction between the sex and genotype (F(1,63) = 1.19, *p* = 0.28, Figure 3B). Regarding eye opening, there was an effect of genotype in the results of eye opening test (F(1,62) = 19.63, *p* < 0.001, Figure 3B). However, there was no effect of sex (F(1,62) = 1.8, *p* = 0.18, Figure 3B) nor an interaction of sex and genotype (F(1,62) = 1.4, *p* = 0.23, Figure 3B). Wild-type males opened their eyes for the first time earlier than *Shank3B*^−/−^ mice of both sexes by approximately 1.5 days. There were no significant differences in the eruption of both lower and upper incisors in *Shank3B*^−/−^ mice. There was no effect of sex (lower incisors: F(1,63) = 0.08, *p* = 0.78, Figure 3C; upper incisors: F(1,63) = 0.45, *p* = 0.51, Figure 3D) or genotype (lower incisors: F(1,63) = 0.40, *p* = 0.53, Figure 3C; upper incisors: F(1,63) = 2.36, *p* = 0.13, Figure 3D).

### 3.3. Reflexes

**Figure 4:**
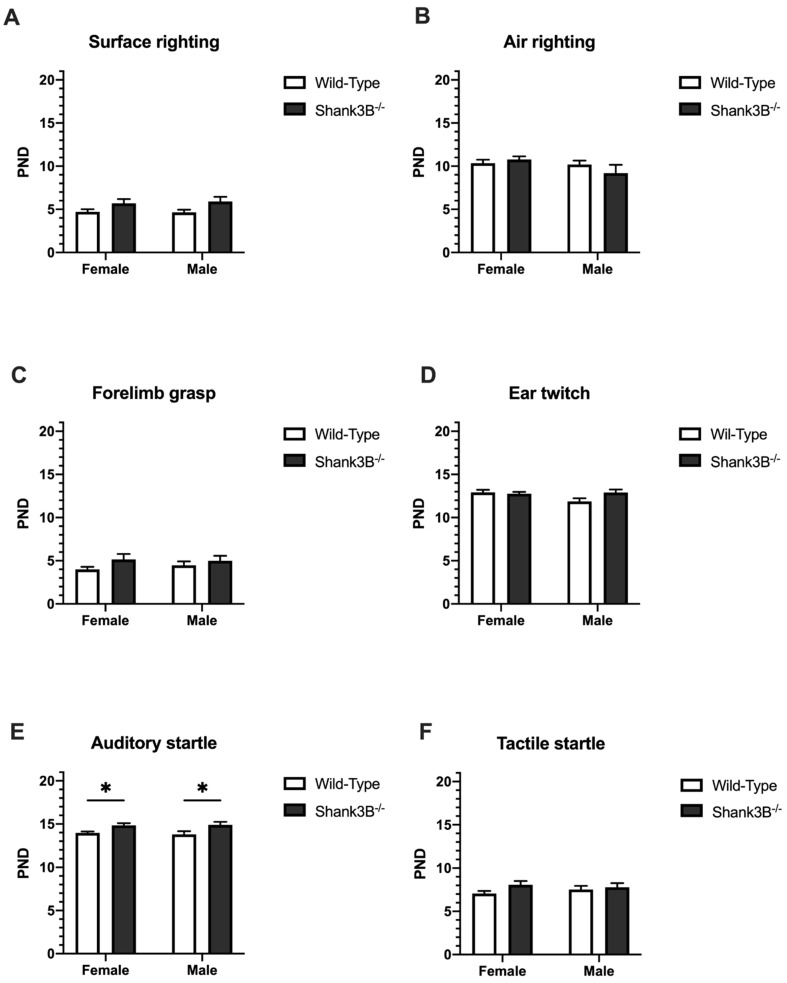
Reflexes. (**A**) Surface righting. (**B**) Air righting. (**C**) Forelimb grasp (**D**) Ear twitch reflex (**E**,**F**) Auditory and tactile startle, respectively. PND = postnatal day; Data are shown as mean + SEM. * denotes *p* < 0.05. *Shank3B*^−/−^: ♀ *n* = 13, ♂ *n* = 10; Wild-Types: ♀ *n* = 29, ♂ *n* = 15.

A significant effect of time (F(4,279 = 284.8, *p* < 0.001) was observed in the surface righting test. During all PNDs, pups from all groups performed equally with gradual improvement, which was comparable among groups in this test. No effect of sex (F(1,62) = 0.02, *p* = 0.88), genotype (F(3,62) = 2.64, *p* = 0.057) or interaction (F(60,1240) = 0.89, *p* = 0.71) was observed (Figure 4A). Similarly, no differences were found in air righting (F(1,63) = 2.387, *p* = 0.13 for genotype; F(1,63) = 0.27, *p* = 0.61 for sex; Figure 4B), and no significant differences in development of other reflexes between *Shank3B*^−/−^ and wild-type mice was observed (forelimb grasp: F(1,63) = 0.11, *p* = 0.74, Figure 4C; ear twitch: F(1,63) = 1.66, *p* = 0.20, Figure 4D; tactile startle: F(1,63) = 0.05, *p* = 0.82, Figure 4F).

However, in the auditory startle, a slight but significant delay by approximately 1 day in response due to the genotype was observed in both females and males (F(1,63) = 12.7, *p* < 0.001; Figure 4E).

### 3.4. Motor Skills

**Figure 5:**
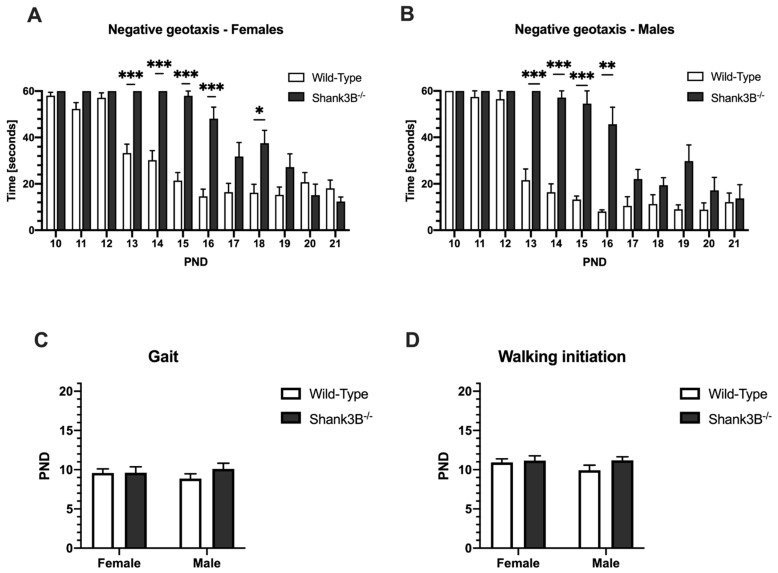
Motor skills. (**A**) Negative geotaxis in females. (**B**) Negative geotaxis in males (**C**,**D**) Gait and walking initiation, respectively. PND = postnatal day; * denotes *p* < 0.05, ** denotes *p* < 0.01 and *** denotes *p* < 0.001. Data are shown as mean + SEM. *Shank3B*^−/−^: ♀ *n* = 13, ♂ *n* = 10; Wild-Types: ♀ *n* = 29, ♂ *n* = 15.

Female *Shank3B*^−/−^ mice were significantly slower at turning and climbing upwards in the negative geotaxis test during the PND = 13–16 than their wild-type litter mates (delay of *Shank3B*^−/−^: t = 26.71; 29.78; 36.58; 33.49 sec respectively, *p* < 0.001, Figure 5A). Male *Shank3B*^−/−^ mice were also significantly slower at turning and climbing upwards in the negative geotaxis test during the PND = 13–15 than their wild-type litter mates (delay of *Shank3B*^−/−^: t = 38.40; 40.73; 41.30 sec respectively, *p* < 0.001, Figure 4B) and also at PND = 16 (*Shank3B*^−/−^: t = 37.50 sec; *p* < 0.01; Figure 5B). There were no detectable differences in the performance of the animal groups in other motor coordination and locomotor activity tests, such as gait (genotype: F(1,62) = 0.82, *p* = 0.37; sex: F(1,62) = 0.03, *p* = 0.86; interaction of genotype and sex: F(1,62) = 0.75, *p* = 0.39, Figure 5C) and walking initiation (genotype: F(1,62) = 1.53, *p* = 0.22; sex: F(1,62) = 0.63, *p* = 0.43; interaction of genotype and sex: F(1,62) = 0.76, *p* = 0.39, Figure 5D).

## 4. Discussion

Our experiment showed that *Shank3B*^−/−^ mice were delayed in eye opening and auditory startle compared to their wild-type littermates. Also, negative geotaxis was impaired between PND 13–16, regardless the gender. Nevertheless, these represent only a few of the measured landmarks, reflexes, or motor skills during early postnatal development of our experiment. According to our knowledge, this is the first thorough examination of early neurodevelopment of potential in an animal ASD model.

Interestingly, *Shank3B*^−/−^ and wild-type mice did not differ in body weight as previously observed by Peça and colleagues, who did not record any discrepancies in body weight of *Shank3*-deficient mice, either [20]. On the other hand, in other rodent models where autism was induced by valproic acid (VPA), or propionic acid (PPA), the body weight of rodents with autistic phenotype was notably lower than that of control animals [21,22]. Thus, it seems a genetically induced model of autism does not seem to manifest by the body weight loss of mutant animals compared to the wild-type controls, whereas those models induced by VPA or PPA do cause weight loss compared to controls, suggesting an important distinction between these two types of ASD models. Regarding the physical and morphological landmarks, we observed that male *Shank3B*^−/−^ open their eyes approximately 1.5 days later than their wild-type litter mates; however, this was not observed in females. There was no significant difference in incisor eruption between the *Shank3B*^−/−^ and wild-type mice. In several other studies, with either genetic (neuroligin-4 null mutant [23], methylenetetrahydrofolate-reductase heterozygous mutant [24]) or environmental (induced by VPA [22,25,26], glucosinate ammonium [27]) animal autistic models, a later eyelid opening was found similarly to our study.

The neonatal developmental period, and corresponding maturation of the central nervous system, was evaluated using a set of tests assessing involuntary reflexes [28,29]. There were no differences between *Shank3B*^−/−^ and wild-type litter mates in observed reflexes. While other genetic animal models of ASD exhibit altered maturation of involuntary reflexes rather consistently [30,31,32], animals with mutations of *Shank3* gene tend to vary. Thus, it is important to take this into consideration when designing a study on pups. On the other hand, a *Shank3* strain with different mutation, *Shank3^Δ4–22^*, has shown significant differences in air righting on PND = 10–12, where the mutant mice were slower than wild-type mice [33]. In the auditory startle, the *Shank3B*^−/−^ female and male mice reacted notably later than their wild-types counterparts. In the mentioned (*Shank3^Δ4–22^*) model, where the mutation of the gene led to disruption of all protein isoforms in comparison to our model, mutant mice were remarkably altered in social behavior as well as motor and sensory behavior compared to wild-type mice [33]. A more complex disruption of SHANK3 protein results in more concordant and replicable behavioral deficits related to autism spectrum disorders.

The negative geotaxis is used to study most neurodevelopmental disorders and diseases of the central nervous system [22,31]. Concerning motor coordination and locomotor activity, *Shank3B*^−/−^ mice were delayed in completing the negative geotaxis test when compared to wild-type mice. Mutant mice took approximately twice as much time reaching the top of the assay, which was observable from PND13 to PND16 for both sexes. Nevertheless, this deficit was diminished by the end of weaning, i.e., PND 21 (Figure 4). The motor coordination delay was observed in other *Shank3* mutant strain (*Shank3^Δ4–22^*) as well as in other models of ASD [22,33,34,35]. The delay in acquisition of postural reflexes could be an important early indicator of motor impairment in this model. To determine if the early deficits in reflex acquisition can predict adult impairments, future studies should focus on the associations of these findings with adult endpoints.

Regarding underlying mechanisms, SHANK3 is a scaffolding protein linking glutamate NMDA and type 1 metabotropic glutamate receptors (mGluR1) to the actin cytoskeleton and enhances the polymerization of actin filaments. With NMDA receptors, SHANK3 is associated via the guanylate kinase-associated protein/postsynaptic density-95 complex [36], and with mGluR1 through the homer protein [37,38]. In the developing brain, SHANK3 is responsible for formation and maturation of dendritic spines, as well as for the morphological spine changes during synaptic plasticity [39,40]. Durand et al. [41] showed that shortening mutations have significantly negative effects on spine development and morphology, as well as growth cone motility [41]. Another study showed that SHANK3-deficient mice had diminished NMDA receptor synaptic function and distribution on the synapses in the prefrontal cortex, as well as loss of actin filament in the cortex. This leads to reduction in activity of Rho GTPase–RAC1, p21-activated kinase, and increased cofilin activity, which contributed to the manifestation of ASD-like phenotypes [42]. Therefore, similar mechanisms may be responsible for the developmental delays and impairments noted in *Shank3B*^−/−^ mice in the current study.

Although ASD is diagnosed early in childhood, most experiments are conducted on adult animals [43,44,45,46].The various animal models of ASD could manifest autistic features such as neuromotor and neurodevelopmental delay predominantly in early stages of development, as is typical in children with ASD [47]. The *Shank3B*^−/−^ model is the most widely used model of ASD, but research into its phenotype throughout neurodevelopment is lacking. Our results show that the neuromotor development of *Shank3B*^−/−^ mice slightly differs in comparison to wild-type mice, regardless of sex. This suggests that the B6.129-*Shank3*^t^^m2Gfng/J^ mutation in *Shank3* gene, with partial gene deletion, is sufficient to cause neurodevelopmental deficits in pups. The observed delays in neonatal development can be more profound when a greater extent of the *Shank3* gene is deleted, as supported by a recent paper on neurodevelopmental milestones assessed in *Shank3^Δ4–22^* mice with complete gene deletion. The mutant mice showed noteworthy sensory-motor deficits and impairment in motor coordination when compared to wild-type mice [33].

In conclusion, our results show that a partial *Shank3B* gene deletion is sufficient to generate several developmental deficits, although a more extensive *Shank3* deletion seem to cause a more profound motor deficits in pups. These motor deficits might be the first sign of ASD, reflective of delayed development in individuals with ASD. However, our study does not bring evidence for an association between neuromotor development deficits and ASD core symptoms, as these were assessed in the current experiment. The lack of major sex differences points towards the several limitations of the used animal model of ASD regarding its applicability to the clinics. The interpretation is complicated also by the relatively low prevalence of Shank3 deficiency among the ASD patients. Further studies are, therefore, needed to evaluate whether the observed motor developmental deficits persist into adulthood, whether behavioral and social deficits appearing later after weaning are linked to these deficits. Most importantly, the observations should be tested in other models of ASD to be of potential use for the wide range of heterogenous entities covered by the umbrella term of ASD.

## Data Availability

The datasets generated during the current study are available from the corresponding author on reasonable request.
